# Latent profiles and influencing factors of sense of coherence in patients with advanced cancer: A cross-sectional study

**DOI:** 10.1016/j.apjon.2025.100848

**Published:** 2025-12-30

**Authors:** Yongqi Huang, Huimin Xiong, Xia Tian, Jinjia Lai, Liqun Zhou, Lili Chen, Wenli Xiao

**Affiliations:** a School of Nursing, Guangzhou University of Chinese Medicine, Guangzhou, Guangdong, China; b Department of Colorectal and Anal Surgery, The First Affiliated Hospital of Guangzhou University of Chinese Medicine, Guangzhou, Guangdong, China; c Oncology Center, The First Affiliated Hospital of Guangzhou University of Chinese Medicine, Guangzhou, Guangdong, China; d Department of Emergency, The First Affiliated Hospital of Guangzhou University of Chinese Medicine, Guangzhou, Guangdong, China

**Keywords:** Sense of coherence, Patients with advanced cancer, Latent profile analysis, Generalized resistance resources, Salutogenesis theory

## Abstract

**Objective:**

This study aims to identify the latent profiles of sense of coherence (SOC) in patients with advanced cancer and explore its influencing factors encompassing sociodemographic and clinical characteristics, and generalized resistance resources (GRRs).

**Methods:**

A cross-sectional study of 262 patients with advanced cancer was conducted by convenience sampling in Guangzhou, China, from September 2023 to July 2024. Data were collected including sociodemographic and clinical characteristics, SOC-13, Revised Life Orientation Test (LOT-R), Rosenberg Self-Esteem Scale (RSES), Inner Peace State Scale (IPSS), Gratitude Questionnaire-6 (GQ-6), and Social Support Rating Scale (SSRS). Statistical analysis was performed using latent profile analysis (LPA) and multivariate logistic regression analysis.

**Results:**

Three latent profiles of SOC were identified: low SOC and low comprehensibility group (29.01%), moderate SOC and high meaningfulness group (40.08%), and high SOC and high manageability group (30.91%). This study found that SOC was impacted by self-perceived severity of the disease and GRRs including optimism, self-esteem, and inner peace (*P* < 0.05).

**Conclusions:**

SOC in patients with advanced cancer exhibited different characteristics. Enhancing positive disease perception and GRRs including optimism, self-esteem, and inner peace may be effective strategies for improving their SOC. Healthcare professionals can formulate strategies such as tailored health education, symptom management, and positive psychological interventions to enhance SOC in patients with advanced cancer.

## Introduction

In 2022, approximately 20 million new cancer diagnoses and 9.7 million deaths were reported worldwide.^1^ Furthermore, by 2050, there will be 35 million new cancer cases predictably, indicating an increasing global cancer burden that needs urgent attention.^1^ While notable advancements have been achieved in medical technology, there remain numerous cancer patients whose condition gradually progresses to advanced stage.^2^ Moreover, due to the delayed presentation of symptoms and diagnosis, some patients encounter difficulty in accepting and coping with the diagnosis of advanced cancer in a short time.

Faced with the complexity of treatment and the uncertainty of cancer progression, patients with advanced cancer experience not only a heavy symptom burden but also psychological distress such as anxiety, fear, demoralization, and ineffective coping.^3^^,^^4^ What’s more, the threat of death presents an additional challenge, which results in a deterioration of their confidence and ability to cope with cancer, and even the thoughts of suicide or hastened death.^5^^,^^6^ Therefore, it is essential to enhance patients’ confidence and ability to manage the challenges presented by advanced cancer, thereby reducing their psychological distress.

In 1979, Aaron Antonovsky originated the salutogenesis theory,^7^ which was closely related to individuals’ stress, health, and coping. It proposed that individuals are moving continuously along the health ease/dis-ease continuum, and sense of coherence (SOC) and generalized resistance resources (GRRs) are key factors that drive individuals to move toward the ease pole,^7^ and cope with stressors effectively.

SOC is the core concept of the salutogenesis theory, defined as a global orientation that expresses a pervasive, enduring, and dynamic sense of confidence.^7^^,^^8^ It reflects the ability of individuals to identify and utilize their internal and external resources to cope effectively with stressors, including three elements: 1) comprehensibility: the stimuli deriving from one’s internal and external environments are structured, predictable, and explicable. 2) manageability: the resources are available to cope with stressors. 3) meaningfulness: the stressors and challenges are worth investment and engagement.^7^^,^^8^ GRRs refer to resources that empower individuals, groups, and communities to cope effectively with stressors, encompassing material resources, knowledge, intelligence, social support, coping strategies, and individuals’ state of mind.^9^

According to the salutogenesis theory, SOC and GRRs exist in a reciprocal and dynamic relationship, where GRRs play a significant role in forming individuals’ SOC, which in turn mobilizes GRRs for effective coping. When GRRs become enduring and integrate into one’s life situation, they are generally regarded as the primary determinants of SOC.^9^ Prior studies have indicated that cancer patients exhibiting higher levels of SOC were more inclined to draw on their GRRs and adopt positive coping strategies to manage the disease.^10^^,^^11^ This allowed them to engage in a positive coping experience, resulting in a higher quality of life. It is thus clear that SOC and GRRs offer a new perspective to improve coping confidence and ability in patients with advanced cancer.

To our knowledge, current studies have commenced to investigate the SOC of patients with advanced cancer.^12^^,^^13^ However, they evaluated the level of SOC simply by total scores, overlooking the possibility that patients may manifest similar total SOC scores while possessing divergent internal characteristics. The dearth of empirical research exploring the heterogeneity of SOC in patients with advanced cancer leads to the difficulty of developing targeted intervention strategies in clinical practice. Latent Profile Analysis (LPA) represents a statistical method that is employed to classify individuals based on their responses to the scale, thereby uncovering distinctive characteristics of various categories.^14^ Compared with traditional cluster analysis, LPA exhibits notable advantages in terms of classification accuracy, statistical rigor, and interpretability of model results.^15^ This approach widely serves as a basis for medical practitioners to identify the distinct psychosocial characteristics of patients, thereby enhancing the precision and efficiency of their practice.

Meanwhile, in our previous qualitative study exploring the disease coping experience of patients with advanced cancer from the perspective of SOC,^16^ we found that positive psychological qualities (including optimism, self-esteem, inner peace, and gratitude) and social support emerged as the most commonly employed GRRs to help them cope with challenges, encompassing symptom burden, psychological distress, and social isolation, etc. However, the impact of these GRRs on SOC requires further verification and analysis. Hence, these GRRs will be incorporated as predictors of various SOC profiles in this study.

Therefore, the present study aimed to: (1) explore the latent profiles of SOC in patients with advanced cancer and (2) analyze influencing factors (sociodemographic and clinical characteristics, GRRs including positive psychological qualities and social support) of distinct SOC profiles, so as to provide new insight into formulating psychological support and coping strategies that are tailored to advanced cancer patients’ psychosocial characteristics and needs.

## Methods

### Study design and participants

A cross-sectional survey was performed in the oncology center of a tertiary Grade-A hospital in Guangzhou, China, from September 2023 to July 2024. Participants were selected by convenience sampling. Patients were included as follows: (1) a malignant tumor diagnosed by pathology, and the disease stage was III or IV, (2) 18 years old and above, and (3) awareness of their disease diagnosis. Patients were excluded as follows: (1) undergoing severe symptoms or complications that made them unable to complete the survey, (2) suffering from severe mental or cognitive disorders.

### Sample size estimation

The primary statistical methods employed in this study were LPA and multivariate logistic regression analysis. According to previous studies,^17^, ^18^, ^19^ LPA needs sample sizes based on the following rules: 1) total samples should have a minimum of 200 cases, 2) each subgroup contained at least 50 samples, and 3) the smallest subgroup comprised more than 5% of the total sample size. At the same time, the required sample size for multivariate logistic regression analysis was calculated using the G∗Power 3.1.9.7 software. The linear multiple regression algorithm was selected, with *α* = 0.05, power (1-β) = 0.8, and an effect value of 0.15. Given that there were 24 independent variables in this study, at least 169 samples need to be collected. The final estimated sample size was 211 cases after combing the requirements of the above-mentioned two statistical methods and taking into account a 20% invalid response rate.

### Procedures

Data collection was completed by three trained researchers. The researchers meticulously reviewed the electronic medical records of patients across the five wards of the oncology center and invited those who met the study criteria to participate. Each participant was informed that their involvement or withdrawal was voluntary. After comprehending the purpose of this study, all participants offered signed informed consent and received a questionnaire in print to obtain their sociodemographic characteristics and responses to the six scales. The questionnaires were filled out anonymously to ensure that participants’ personal information was not disclosed. Questionnaire completion took about 15–30 min. If there is a significant change in the patient’s condition or emotion, the survey will be stopped to ensure the patient’s safety and the accuracy of the data. In instances of response fatigue, participants were given the option of either continuing after a brief respite or withdrawing from the study. At the time of retrieval, the researchers checked the questionnaire carefully and returned it to the participants if incomplete and inconsistent responses were found. Participants’ clinical characteristics were collected from medical records.

### Measures

#### Sociodemographic and clinical characteristics

Information including age, gender, educational level, marital status, occupation status, residence, religious belief, type of caregiver, self-perceived financial stress, self-perceived severity of the disease, cancer site, stage, anti-tumor treatment types, and disease duration were collected.

#### Sense of coherence

SOC-13 was developed by Antonovsky^20^ and revised by Bao et al.^21^ The scale contains three dimensions, including comprehensibility (5 items), meaningfulness (4 items), and manageability (4 items). A scoring method employing a 7-point Likert scale is implemented. The quartiles of SOC total scores in this study are as follows: P_25_ = 56, P_50_ = 64, and P_75_ = 73, and the range of total scores from 13 to 56, 57–72, and 73–91 indicate low, moderate, and high SOC levels, respectively. The Cronbach’s alpha coefficients for the original SOC-13 exhibited a range of 0.74–0.91,^20^ while the Chinese version yielded a coefficient of 0.76.^21^ In this study, the Cronbach’s alpha coefficient for the SOC-13 was 0.824.

#### Optimism

The Revised Life Orientation Test (LOT-R) was employed to measure the optimistic traits in individuals with 6 items. This scale was developed by Scheier et al.^22^ and revised in Chinese by Lai et al.^23^ A 5-point Likert scale scoring system is used, ranging from 0 (strongly disagree) to 4 (strongly agree). The higher total scores range from 0 to 24, reflecting a greater tendency toward optimism in one’s life. The Cronbach’s alpha coefficient for the Chinese version of LOT-R was 0.70.^23^ The Cronbach’s alpha coefficient for this study sample was 0.524.

#### Self-esteem

Rosenberg Self-Esteem Scale (RSES) was developed by Rosenberg^24^ with 10 items. A 4-point Likert scale scoring methodology is employed (1 = strongly disagree, 4 = strongly agree). Ranging from 10 to 40, a higher total score of RSES indicates a higher level of self-esteem in individuals. Ji et al.^25^ revised the Chinese version of RSES, resulting in a Cronbach’s alpha coefficient of 0.82. The Cronbach’s alpha coefficient in this study was 0.847.

#### Inner peace

The Inner Peace State Scale (IPSS) originally developed by Wang et al.,^26^ was designed to evaluate individuals’ inner peace in life. IPSS consists of 7 items, all of which are scored on a 5-point Likert scale (0 = not at all, 4 = very much). The total score is evaluated on a range of 0–28, with higher scores showing greater inner peace. The Cronbach’s alpha coefficient of IPSS was 0.89.^26^ In this study, the Cronbach’s alpha coefficient for the IPSS was 0.881.

#### Gratitude

The Gratitude Questionnaire-6 (GQ-6), a short scale utilized to evaluate individuals’ gratitude traits, was developed by McCullough et al.^27^ It contains 6 items, each of which adopts a 7-point Likert scale for scoring, with 1 indicating strongly disagree and 7 indicating strongly agree. Ranging from 6 to 42, the higher total scores demonstrate a stronger gratitude tendency. Wei et al.^28^ revised the GQ-6 in Chinese and reported a Cronbach’s alpha coefficient of 0.81. The Cronbach’s alpha coefficient for this study sample was 0.664.

#### Social support

Social Support Rating Scale (SSRS) was a 10-item measurement tool developed by Xiao,^29^ which consists of 3 dimensions to assess the levels of individuals’ objective support, subjective support, and utilization of social support. Ranging from 12 to 66, the higher total scores on the scale exhibit a greater adequacy of social support. The scale has been widely utilized within the Chinese population, and its Cronbach’s alpha coefficients have been reported to range from 0.89 to 0.94.^29^ In this study, the Cronbach’s alpha coefficient for the SSRS was 0.647.

### Data analysis

Mplus 8.3 and SPSS 26.0 software were used to analyze the data. Statistical significance was set at *P* < 0.05. First, LPA was performed based on the scores of the 13 items of the SOC-13 by Mplus 8.3 software. The model categories were gradually increased from a single one, and the model fitting criteria included:^14^ 1) Akaike information criterion (AIC), Bayesian information criterion (BIC), and adjusted Bayesian information criterion (aBIC). Smaller values represented superior model fit. 2) Entropy. When Entropy value ≥ 0.8, it indicated that the classification accuracy was higher than 90%. 3) Lo-Mendell–Rubin test (LMR) and Bootstrap likelihood ratio test (BLRT), were utilized to compare the models. When the *P*-values of both were significant, showing that the k class model outperformed the k-1 class model. The best model categories were finally selected by combining the fitting criteria and practical implications. Second, a descriptive analysis was performed by SPSS 26.0 software. Sociodemographic and clinical characteristics were mainly described using frequency and percentage, while continuous variables including age, SOC, and GRRs (normal distribution and homogeneity of variance in this study) were represented by mean ± standard deviation (SD). The differences in the potential profiles of SOC in sociodemographic and clinical characteristics, as well as GRRs, were analyzed using the χ^2^ test or one-way analysis of variance (ANOVA). Finally, multivariate logistic regression analysis was conducted to analyze the factors that influence the latent profiles of SOC. The variance inflation factor (VIF) values for the independent variables were all less than 5 in this study, indicating that no multicollinearity existed among those variables.

## Results

### Characteristics of participants

**Fig. 1:**
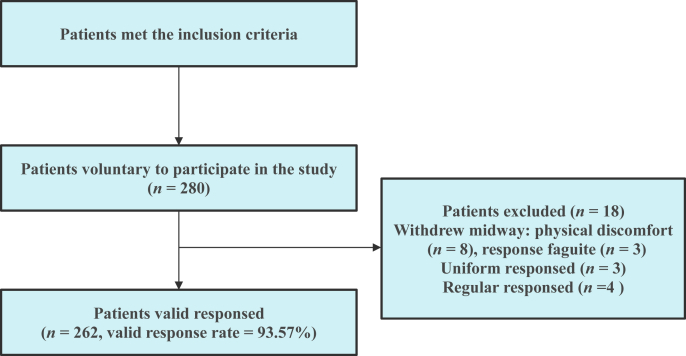
Participants flow diagram.

Two hundred and eighty patients were included in the study, and 262 participants completed it, indicating a valid response rate of 93.57%. Eighteen participants were excluded due to incomplete, uniform, or regular responses (Fig. 1). The mean age of the participants was 52.16 years (SD = 10.82). We found that 49.62% of participants were male and 10.69% had a primary school education or below. Nearly 90.84% of participants were married and 28.24% were employed. Also, 67.56% of participants resided in urban areas and 13.36% reported having a religious belief. Most of the caregivers were spouses (52.29%). Furthermore, 22.90% of participants indicated no or low financial stress, and 19.08% perceived the severity of their own disease as mild. Regarding clinical characteristics, 41.60% of participants were diagnosed with cancer of the digestive system and most (77.48%) were in stage IV of the disease. Additionally, 11.45% of participants had no clear treatment plan, and 35.88% had a disease duration of ≤ 6 months (Table 1).

**Table 1:** Differences in Sociodemographic and clinical characteristics among the latent profiles of SOC (*N* = 262).

Variables	All samples, *n* (%) (*n* = 262, 100.00%)	Class 1, *n* (%) (*n* = 76, 29.01%)	Class 2, *n* (%) (*n* = 105, 40.08%)	Class 3, *n* (%) (*n* = 81, 30.91%)	Statistical values	*P* value
**Age (M ± SD)**	52.16 ± 10.82	49.22 ± 10.48	52.97 ± 11.08	53.88 ± 10.34	4.218^a^	0.016^c^
**Sex**					7.006^b^	0.030^c^
Male	130 (49.62)	28 (21.54)	58 (44.62)	44 (33.85)		
Female	132 (50.38)	48 (36.36)	47 (35.61)	37 (28.03)		
**Educational level**					14.091^b^	0.079
Primary school and below	28 (10.69)	10 (35.71)	9 (32.14)	9 (32.14)		
Junior high school	93 (35.50)	33 (35.48)	40 (43.01)	20 (21.51)		
Senior high school	67 (25.57)	21 (31.34)	22 (32.84)	24 (35.82)		
Junior college	38 (14.50)	8 (21.05)	15 (39.47)	15 (39.47)		
Bachelor degree and above	36 (13.74)	4 (11.11)	19 (52.78)	13 (36.11)		
**Marital status**					8.285^b^	0.016^c^
Unmarried / Divorced / Widowed	24 (9.16)	13 (54.17)	7 (29.17)	4 (16.67)		
Married	238 (90.84)	63 (26.47)	98 (41.18)	77 (32.35)		
**Occupation status**					4.127^b^	0.389
Employed	74 (28.24)	25 (33.78)	28 (37.84)	21 (28.38)		
Unemployed	80 (30.53)	27 (33.75)	30 (37.50)	23 (28.75)		
Retired	108 (41.22)	24 (22.22)	47 (43.52)	37 (34.26)		
**Residence**					8.724^b^	0.013^c^
Urban area	177 (67.56)	46 (25.99)	66 (37.29)	65 (36.72)		
Rural area	85 (32.44)	30 (35.29)	39 (45.88)	16 (18.82)		
**Religious belief**					2.265^b^	0.322
Yes	35 (13.36)	12 (34.29)	16 (45.71)	7 (20.00)		
No	227 (86.64)	64 (28.19)	89 (39.21)	74 (32.60)		
**Type of caregiver**					9.152^b^	0.165
Spouse	137 (52.29)	34 (24.82)	51 (37.23)	52 (37.96)		
Parents / Children	65 (24.80)	24 (36.92)	28 (43.08)	13 (20.00)		
Others	9 (3.44)	3 (33.33)	5 (55.56)	1 (11.11)		
None	51 (19.47)	15 (29.41)	21 (41.18)	15 (29.41)		
**Self-perceive financial stress**					30.214^b^	< 0.001^d^
None / Low	60 (22.90)	7 (11.67)	19 (31.67)	34 (56.67)		
Middle	97 (37.02)	29 (29.9)	39 (40.21)	29 (29.90)		
High	105 (40.08)	40 (38.1)	47 (44.76)	18 (17.14)		
**Self-perceived severity of the disease**					31.751^b^	< 0.001^d^
Mild	50 (19.08)	6 (12.00)	15 (30.00)	29 (58.00)		
Moderate	71 (27.10)	18 (25.35)	26 (36.62)	27 (38.03)		
Severe	141 (53.82)	52 (36.88)	64 (45.39)	25 (17.73)		
**Cancer site**					10.743^b^	0.378
Digestive system	109 (41.60)	35 (32.11)	38 (34.86)	36 (33.03)		
Lung	75 (28.63)	19 (25.33)	33 (44.00)	23 (30.67)		
Nasopharynx	26 (9.92)	7 (26.92)	10 (38.46)	9 (34.62)		
Gynecological system	25 (9.54)	10 (40.00)	7 (28.00)	8 (32.00)		
Breast	16 (6.11)	2 (12.50)	11 (68.75)	3 (18.75)		
Others	11 (4.20)	3 (27.27)	6 (54.55)	2 (18.18)		
**Cancer stage**					0.922^b^	0.631
Ⅲ	59 (22.52)	17 (28.81)	21 (35.59)	21 (35.59)		
Ⅳ	203 (77.48)	59 (29.06)	84 (41.38)	60 (29.56)		
**Anti-tumor treatment types (chemotherapy / Radiotherapy / Targeted therapy / Immunotherapy)**					0.946^b^	0.988
None	30 (11.45)	9 (30.00)	12 (40.00)	9 (30.00)		
One type	100 (38.17)	29 (29.00)	38 (38.00)	33 (33.00)		
Two types	115 (43.89)	32 (27.83)	48 (41.74)	35 (30.43)		
Three types and above	17 (6.49)	6 (35.29)	7 (41.18)	4 (23.53)		
**Disease duration**					8.334^b^	0.215
≤ 6 months	94 (35.88)	25 (26.60)	37 (39.36)	32 (34.04)		
> 6 months, ≤ 1 year	34 (12.98)	6 (17.65)	15 (44.12)	13 (38.23)		
> 1 year, ≤ 2 years	54 (20.61)	23 (42.59)	20 (37.04)	11 (20.37)		
> 2 years	80 (30.53)	22 (27.50)	33 (41.25)	25 (31.25)		

SOC, sense of coherence; M, mean; SD, standard deviation.

Class 1: Low SOC and low comprehensibility group; Class 2: Moderate SOC and high meaningfulness group; Class 3: High SOC and high manageability group.

a The one-way ANOVA.

b χ^2^ test.

c *P* < 0.05.

d *P* < 0.001.

### LPA of participants

A total of four models were fitted (Table 2). The values of AIC, BIC, and aBIC showed a gradual declining tendency as the number of fitting models increased, meanwhile, the entropy values exceeded 0.8, indicating that the models were well-fitted and the classification results were reliable. When fitting to the fourth category, the result of the LMR was not significant (*P* = 0.202), suggesting that the third model was better than it. Moreover, each subgroup contained more than 50 samples, and each category had a probability of more than 5%, meeting the sample size requirements for LPA. The SOC of patients with advanced cancer was ultimately categorized into three latent profiles based on the model’s fit indices and clinical implications.

**Table 2:** Latent profile analysis models and fit indices of SOC (*n* = 262).

Model	k	AIC	BIC	aBIC	Entropy	LMR	BLRT	Classes probability
1	26	12,193.932	12,286.709	12,204.277	–	–	–	–
2	40	11,614.735	11,757.469	11,630.651	0.860	< 0.001^b^	< 0.001^b^	41.22% / 58.78%
**3**	**54**	**11,479.370**	**11,672.060**	**11,500.856**	**0.828**	**0.019**^a^	**< 0.001**^b^	**29.01% / 40.08% / 30.91%**
4	68	11,386.115	11,628.762	11,413.172	0.891	0.202	< 0.001^b^	27.86% / 27.48% / 33.59% / 11.07%

AIC, Akaike information criterion; BIC, Bayesian information criterion; aBIC, adjusted Bayesian information criterion; LMR, Lo-Mendell–Rubin test; BLRT, Bootstrap likelihood ratio test; SOC, sense of coherence.

a *P* < 0.05.

b *P* < 0.001.

**Fig. 2:**
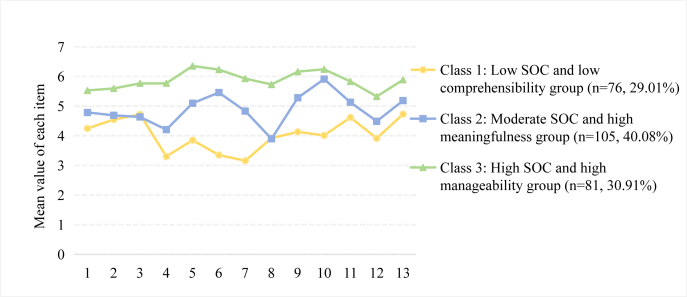
Characteristics of the latent profiles of SOC in patients with advanced cancer. SOC, sense of coherence.

According to our findings, there were three distinct latent profiles of SOC among participants (Fig. 2). The naming of SOC latent profiles was derived from the aggregate SOC scores and their distinct dimensional subscale performances. Class 1, comprising 76 individuals (29.01%), exhibited the lowest level of SOC, as indicated by a mean SOC-13 score of 52.28 (SD = 6.09). Moreover, the mean score of items in the comprehensibility dimension was the lowest. Therefore, it was classified as the “low SOC and low comprehensibility group”. Class 2 consisted of 105 participants (40.08%), who demonstrated a moderate level of SOC, with a mean SOC-13 score of 63.46 (SD = 4.76) and the highest mean score of items in the meaningfulness dimension, so this group was designated as the “moderate SOC and high meaningfulness group”. Class 3 composed of 81 individuals (30.91%), and had the highest level of SOC, with a mean SOC-13 score of 76.70 (SD = 4.36) and the highest mean score of items in the manageability dimension, thus was named the “high SOC and high manageability group”.

### Univariate analysis of the SOC latent profiles

The univariate analysis (Table 1) indicated significant differences in age, gender, marital status, residence, self-perceived financial stress, and self-perceived severity of the disease (*P* < 0.05) among the three latent profiles of SOC in patients with advanced cancer. According to the one-way ANOVA (Table 3), there existed statistical significance in the total scores of optimism, self-esteem, inner peace, gratitude, and social support among different profiles (*P* < 0.001).

**Table 3:** Comparison of optimism, self-esteem, inner peace, gratitude and social support among the latent profiles of SOC (*N* = 262).

Variables	Class 1 (*n* = 76) M ± SD	Class 2 (*n* = 105) M ± SD	Class 3 (*n* = 81) M ± SD	*F*	*P* value	Comparison
**Sense of coherence**	52.28 ± 6.09	63.46 ± 4.76	76.70 ± 4.36	458.085	< 0.001^a^	C1 < C2 < C3
Comprehensibility	18.82 ± 3.33	23.27 ± 2.67	28.99 ± 2.10	275.906	< 0.001^a^	C1 < C2 < C3
Manageability	15.80 ± 2.98	19.86 ± 3.04	24.19 ± 2.43	170.096	< 0.001^a^	C1 < C2 < C3
Meaningfulness	17.66 ± 2.91	20.33 ± 2.95	23.53 ± 2.34	89.124	< 0.001^a^	C1 < C2 < C3
**Optimism**	16.59 ± 2.95	17.13 ± 2.41	18.75 ± 2.30	15.715	< 0.001^a^	C1 = C2 < C3
**Self-esteem**	29.43 ± 3.98	30.36 ± 3.58	33.70 ± 3.28	31.342	< 0.001^a^	C1 = C2 < C3
**Inner peace**	16.01 ± 4.76	19.08 ± 4.42	22.95 ± 3.32	53.480	< 0.001^a^	C1 < C2 < C3
**Gratitude**	32.46 ± 4.68	32.71 ± 4.85	35.72 ± 4.69	13.528	< 0.001^a^	C1 = C2 < C3
**Social support**	40.72 ± 6.96	41.92 ± 6.46	46.32 ± 6.52	16.013	< 0.001^a^	C1 = C2 < C3

SOC, sense of coherence; M, mean; SD, standard deviation.

Class 1: Low SOC and low comprehensibility group; Class 2: Moderate SOC and high meaningfulness group; Class 3: High SOC and high manageability group.

a *P* < 0.001.

### Multivariate logistic regression analysis of the SOC latent profiles

Multivariate logistic regression analysis was employed to observe the factors that influenced distinct latent profiles of SOC (Table 4). The “high SOC and high manageability group” was set as the reference group, with significant variables identified in the univariate analysis serving as independent predictors, while considering the SOC profiles as the dependent variables. The results showed that participants exhibiting high levels of optimism (*OR* = 0.804, *P* = 0.028), self-esteem (*OR* = 0.859, *P* = 0.044), and inner peace (*OR* = 0.730, *P* < 0.001) were more likely to be classified into the “high SOC and high manageability group” in comparison to the “low SOC and low comprehensibility group”. Additionally, participants who perceived the severity of the disease as mild (*OR* = 0.309, *P* = 0.014), and demonstrated high levels of optimism (*OR* = 0.829, *P* = 0.034) and inner peace (*OR* = 0.828, *P* < 0.001) were more likely to be placed in the “high SOC and high manageability group” when compared to the “moderate SOC and high meaningfulness group”.

**Table 4:** Multivariate logistic regression analysis of the influencing factors of the latent profiles of SOC (*N* = 262).

Variables	Class 1 vs. Class 3				Class 2 vs. Class 3			
	*β*	*P* value	OR	95% CI	*β*	*P* value	OR	95% CI
**Age**	−0.037	0.091	0.964	0.924–1.006	−0.015	0.416	0.985	0.950–1.022
Sex								
Male	−0.729	0.105	0.482	0.200–1.166	−0.099	0.797	0.906	0.426–1.925
Female	Ref							
**Marital status**								
Unmarried/ Divorced /Widowed	0.939	0.275	2.557	0.473–13.809	−0.041	0.961	0.960	0.188–4.908
Married	Ref							
**Residence**								
Urban area	−0.671	0.198	0.511	0.184–1.419	−0.634	0.165	0.530	0.216–1.299
Rural area	Ref							
**Self-perceive financial stress**								
None / Low	−1.227	0.063	0.293	0.081–1.066	−0.708	0.156	0.493	0.185–1.310
Middle	−0.148	0.780	0.863	0.306–2.434	−0.263	0.575	0.769	0.307–1.927
High	Ref							
**Self-perceived severity of disease**								
Mild	−1.205	0.053	0.300	0.088–1.015	−1.173	**0.014**^a^	0.309	0.122–0.787
Moderate	−0.726	0.168	0.484	0.173–1.357	−0.707	0.118	0.439	0.203–1.197
Severe	Ref							
**Optimism**	−0.218	**0.028**^a^	0.804	0.662–0.977	−0.188	**0.034**^a^	0.829	0.696–0.986
**Self-esteem**	−0.152	**0.044**^a^	0.859	0.741–0.996	−0.112	0.083	0.894	0.787–1.015
**Inner peace**	−0.314	**< 0.001**^b^	0.730	0.648–0.824	−0.189	**< 0.001**^b^	0.828	0.745–0.920
**Gratitude**	−0.001	0.987	0.999	0.894–1.117	−0.014	0.777	0.986	0.894–1.088
**Social support**	−0.065	0.096	0.937	0.868–1.012	−0.056	0.088	0.945	0.886–1.008

SOC, sense of coherence; CI, confidence interval; OR, odds ratio. Class 1: Low SOC and low comprehensibility group; Class 2: Moderate SOC and high meaningfulness group; Class 3: High SOC and high manageability group.

a *P* < 0.05.

b *P* < 0.001.

## Discussion

This study identified the characteristics and influencing factors of SOC in patients with advanced cancer, which enriches our comprehension of SOC in this patient population and guides us in formulating tailored intervention strategies.

The present study revealed that patients with advanced cancer exhibited certain heterogeneity in their SOC, which could be classified into three latent profiles: low SOC and low comprehensibility group (29.01%), moderate SOC and high meaningfulness group (40.08%), and high SOC and high manageability group (30.91%). Liu’s research^30^ concerning cancer radiotherapy patients and Wang’s study^31^ among caregivers of breast cancer patients also identified three latent profiles of SOC, but the characteristics significantly differed from those observed in this study. Additionally, the proportion of individuals in the “low SOC group” in this study was higher than in those two studies. Therefore, it is emphasized the importance of considering not only the total scores on the SOC scale but also the specific responses of individuals when developing tailored intervention strategies for patients with advanced cancer.

Patients in the low SOC and low comprehensibility group may be more inclined to perceive cancer as a huge stressor and demonstrate difficulty in understanding and accepting the cancer diagnosis, as well as coping with the distress caused by cancer, resulting in a lower SOC. Based on the needs and comprehension levels of patients, healthcare professionals may provide individual or group step-by-step health education concerning the incidence, development, treatment, and symptom management of cancer. These education contents can be in the form of lectures, brochures, or electronic media, with the aim of fostering an accurate disease perception and enhancing their SOC.

The moderate SOC and high meaningfulness group represented a significant proportion of the total population. According to the salutogenesis theory,^8^ the dimension of meaningfulness, as the most important element of the SOC may serve as a source of motivation for patients to pursue available resources and cope with cancer. Confronted with a diagnosis of advanced cancer, patients in this group may derive motivation to persevere in their struggle against cancer by acknowledging the meaning of coping with the disease, which encompassed perceived disease benefits and personal growth. Consequently, medical staff may consider employing positive self-disclosure^32^ and meaning therapy^33^ as strategies to help patients find and reinforce their sense of meaning, such as encouraging patients to disclose their disease story, celebrating their achievements in coping with the cancer, and establishing new life goals and treatment expectations, thus promoting the motivation to continue living.

Individuals categorized within the high SOC and high manageability group manifested the greatest amount of SOC, indicating their pronounced ability to manage stressors effectively. Moreover, patients in this group possess higher levels of GRRs than the other groups in this study. Faced with diverse challenges stemming from cancer diagnosis and treatment, they were more likely to demonstrate proficiency in perceiving and mobilizing available GRRs to effectively manage the threats posed by the disease and avoid further physical and psychological distress. Hence, healthcare providers are expected to affirm the coping abilities and strategies of individuals in this group. It may be beneficial to encourage patients to maintain a healthy lifestyle, actively participate in medical decision-making, and self-manage symptoms, which aims to gain a sense of control over their lives.^34^ Furthermore, encouraging individuals in this group to share their coping experiences with fellow patients may collectively enhance their SOC levels.

We found that patients with mild self-perceived severity of the disease were more prone to be classified into the high SOC and high manageability group than the moderate SOC and high meaningfulness group. It may be because that individuals may exhibit a more positive disease perception, demonstrating an ability to utilize their resources and knowledge to rapidly comprehend and accept the disease. Furthermore, they tend to adopt a more positive mindset and approach, such as adopting healthy behaviors, to effectively cope with the disease.^32^^,^^35^ Additionally, patients who perceive their disease severity as mild may suffer from fewer symptoms, which affords them sufficient energy and confidence to cope with stressors.^36^ Thus, in addition to aiding patients with advanced cancer in building a positive disease perception, healthcare practitioners should facilitate the implementation of dynamic and effective symptom management strategies. This allows patients to acquire essential health information and skills to properly manage cancer, thereby alleviating the uncertainty and fear of cancer progression.^2^^,^^37^

Our findings indicated that patients with advanced cancer who exhibited higher levels of GRRs, including optimism, self-esteem, and inner peace, were more inclined to belong to the high SOC and high manageability group. The diagnosis and treatment of cancer result in progressive alterations to patients’ body functions and self-image, which substantially influence their overall quality of life. Some patients may encounter numerous negative emotions, including stigma, death anxiety, and demoralization, which ultimately contribute to a loss of confidence in life.^6^^,^^38^ Previous studies indicated that patients who demonstrated pronounced optimism and SOC tend to manifest enhanced disease resilience.^39^ Individuals with higher optimism may be full of expectations for life and be able to make psychological adjustments quickly after experiencing the shock of a cancer diagnosis. Hence, they tend to view cancer as one of the challenges in life with a positive attitude and utilize diverse strategies to alleviate the negative influences caused by cancer effectively.^40^ A study by Cheng et al.^41^ indicated that the spiritual needs of Chinese patients with advanced cancer included “being treated as normal and independent individuals” and “seeking inner peace”. Patients exhibiting greater self-esteem tend to possess more positive self-perceptions and self-evaluations and believe that they can deal with the challenges successfully, which reduces their death anxiety and fear of cancer recurrence.^42^ Influenced by traditional Chinese culture, Chinese people advocate taking things as they come and pursuing inner peace and harmony. Patients who had attained a state of inner peace were more inclined to accept cancer calmly and mobilize available resources to cope with the disease. This enables them to overcome the challenges posed by cancer and recover quickly from its negative impacts, thus maintaining a balance between the management of the disease and the enjoyment of life.^16^^,^^41^ Furthermore, Cecon’s study revealed that lower levels of GRRs and SOC exhibited more psychological care needs in cancer patients.^43^ Therefore, healthcare practitioners can implement positive psychological interventions, such as acceptance and commitment therapy and dignity therapy,^44^ thus improving the positive psychological qualities, cognitions, and behaviors of patients with advanced cancer, and ultimately enhancing their SOC.

Additionally, although gratitude and social support were recognized as important GRRs in patients with advanced cancer,^16^^,^^45^ our findings indicated that their impact on SOC was not significant. This may be attributed to the participants in this study experienced more positive emotions and received various external support from medical staff and family during their hospitalization. This resulted in relatively high levels of gratitude and social support in all SOC profiles, so further multi-center investigations are warranted to explore their influences on distinct profiles of SOC.

According to the salutogenesis theory, SOC and GRRs function as pivotal factors in health promotion. SOC may exert a potential inverse effect on GRRs, indicating that the stronger the SOC among patients with advanced cancer, the greater the GRRs they are likely to acquire. The salutogenesis theory explains why some individuals maintain healthy despite exposure to stressors (due to higher levels of SOC and GRRs). However, it offers limited insight into how individuals dynamically act to address challenges, which is precisely the core of stress and coping theory.^46^ Future integration of these complementary frameworks may facilitate a more comprehensive understanding of the coping mechanisms and processes experienced by patients with advanced cancer.

## Implications for nursing practice and research

Evaluating the characteristics of SOC in patients with advanced cancer is crucial for understanding their confidence and ability in the process of coping with the disease. This study indicated that patients in the low SOC and low comprehensibility group typically exhibit lower cognitive comprehension and GRRs. Early identification of this group, coupled with targeted interventions, may facilitate their acceptance and response to cancer, thereby alleviating their psychological distress. This highlights the significance of assessing the divergent internal characteristics of SOC and fostering positive disease perception and GRRs. Furthermore, positive psychological qualities such as optimism, self-esteem, and inner peace significantly contribute to the growth of SOC in patients with advanced cancer. Hence, medical staff should strengthen interdisciplinary cooperation and training. Based on the latent profiles, influencing factors of SOC, and patients’ cognitive and cultural level, integrating positive psychology with the salutogenesis theory to develop tailored intervention strategies (such as group form of psychological intervention) at different stages of disease progression may prove a possible approach to assisting patients’ flexible psychological adjustment, thus precisely improving their positive emotions and SOC.

## Limitations

This study presents certain limitations. First, it was conducted in only one hospital in China, which may cause the limitation of the external validation and generalizability of the findings to patients with advanced cancer from different countries and cultures. Second, although researchers utilized uniform language to explain the study to patients prior to the survey and strictly control the quality of data collection, self-reported outcomes may have been influenced by cognitions, emotions, and external factors, resulting in a certain degree of measurement bias. A convenience sampling method may cause selection bias and potential unmeasured confounding variables may be present. Also, patients with advanced cancer in critical condition were unable to participate in the survey, possibly resulting in a paucity of understanding regarding their psychological state. Thirdly, LPA may have the limitation of potential model overfitting. Additionally, the types of GRRs in this study were derived from our previous qualitative study, and there may be other types (such as coping style, resilience, spirituality) influencing SOC that warrant further studies. Different GRRs variables may have potential overlap and interaction effects that should be explored in the future. Furthermore, it may be difficult to fully capture the psychological experience of patients with advanced cancer by using a scale to evaluate complex psychological structures such as SOC. Therefore, future studies could consider combining positive psychology and the salutogenesis theory, and conducting multi-center mixed methods research or longitudinal studies based on their disease progression and treatment cycles to explore more types of GRRs and their potential impact mechanism on SOC in patients with advanced cancer, even the change trajectories of these variables, thus providing more possible paths for enhancing their SOC.

## Conclusions

The SOC of patients with advanced cancer was divided into three profiles: low SOC and low comprehensibility group, moderate SOC and high meaningfulness group, and high SOC and high manageability group. These profiles were significantly impacted by the self-perceived severity of the disease, optimism, self-esteem, and inner peace. These findings have provided new insights for healthcare professionals to fully recognize the heterogeneity of SOC. Future research may formulate tailored cancer-related health education, symptom management, and positive psychological interventions based on our findings to enhance the SOC in patients with advanced cancer, which may gradually improve their confidence and capability to cope with cancer, as well as promote their mental health.

## CRediT authorship contribution statement

**Yongqi Huang**: Conceptualization, Methodology, Investigation, Formal analysis, and Writing-original draft. **Huimin Xiong**: Investigation, Writing-review and editing. **Xia Tian**: Investigation, Writing-review and editing. **Jinjia Lai**: Formal analysis, Writing-review and editing. **Liqun Zhou**: Methodology, Writing-review and editing. **Lili Chen**: Methodology, Writing-review and editing. **Wenli Xiao**: Conceptualization, Methodology, Supervision, Writing-review and editing. All authors reviewed and approved the final version of the manuscript.

## Ethics statement

This study was approved by the Ethics Committee of the First Affiliated Hospital of Guangzhou University of Chinese Medicine (Approval No. k-2023-109) and was conducted in accordance with the 1964 Helsinki Declaration and its later amendments or comparable ethical standards. All participants provided written informed consent.

## Data availability statement

The data that support the findings of this study are available from the corresponding author upon reasonable request.

## Declaration of Generative AI and AI-assisted technologies in the writing process

No AI tools/services were used during the preparation of this work.

## Funding

This study was supported by funding from the 2024 Humanities and Social Sciences Research Youth Fund Project of the Ministry of Education (Grant No. 24YJC840038). The funders had no role in considering the study design or in the collection, analysis, interpretation of data, writing of the report, or decision to submit the article for publication.

## Declaration of competing interest

The authors declare no conflict of interest.

## References

[1] Bray F., Laversanne M., Sung H. (2024). Global cancer statistics 2022: GLOBOCAN estimates of incidence and mortality worldwide for 36 cancers in 185 countries. Ca Cancer J Clin.

[2] Yang L., Liu J., Liu Q. (2023). The relationships among symptom experience, family support, health literacy, and fear of progression in advanced lung cancer patients. J Adv Nurs.

[3] Obispo B., Cruz-Castellanos P., Fernandez-Montes A. (2023). Coping strategies as mediators of uncertainty and psychological distress in patients with advanced cancer. Psychooncology.

[4] Huda N., Shaw M.K., Chang H. (2022). Psychological distress among patients with advanced cancer: a conceptual analysis. Cancer Nurs.

[5] Belar A., Martinez M., Centeno C. (2021). Wish to die and hasten death in palliative care: a cross-sectional study factor analysis. Bmj Support Palliat.

[6] Hong Y.T., Lin Y.A., Pan Y.X. (2022). Understanding factors influencing demoralization among cancer patients based on the bio-psycho-social model: a systematic review. Psychooncology.

[7] Antonovsky A. (1987).

[8] Antonovsky A. (1996). The salutogenic model as a theory to guide health promotion. Health Promot Int.

[9] Idan O., Eriksson M., Al-Yagon M., Mittelmark M.B., Bauer G.F., Vaandrager L. (2022). The Handbook of Salutogenesis.

[10] Zamanian H., Amini-Tehrani M., Jalali Z. (2022). Stigma and quality of life in women with breast cancer: mediation and moderation model of social support, sense of coherence, and coping strategies. Front Psychol.

[11] Kim H.S., Nho J., Nam J. (2021). A serial multiple mediator model of sense of coherence, coping strategies, depression, and quality of life among gynecologic cancer patients undergoing chemotherapy. Eur J Oncol Nurs.

[12] Möllerberg M., Årestedt K., Swahnberg K. (2019). Family sense of coherence and its associations with hope, anxiety and symptoms of depression in persons with cancer in palliative phase and their family members: a cross-sectional study. Palliat Med.

[13] Cui P., Shi J., Li S. (2023). Family resilience and its influencing factors among advanced cancer patients and their family caregivers: a multilevel modeling analysis. BMC Cancer.

[14] Ferguson S.L.,G., Moore E.W., Hull D.M. (2020). Finding latent groups in observed data: a primer on latent profile analysis in Mplus for applied researchers. Int J Behav Dev.

[15] Hu M., A T., Zhang X. (2025). Attitudes toward seeking professional psychological help among patients with colorectal cancer: a latent profile analysis. Asia Pac J Oncol Nurs.

[16] Huang Y., Tian X., Wang Z. (2024). Disease coping experience of patients with advanced cancer from the perspective of sense of coherence: a qualitative study. Chin J Nurs.

[17] Hu C., Weng Y., Wang Q. (2024). Fear of progression among colorectal cancer patients: a latent profile analysis. Support Care Cancer.

[18] Yang Y., Lu J., Dong Y. (2025). A latent profile and network analysis of social isolation in colorectal cancer patients undergoing chemotherapy. Asia Pac J Oncol Nurs.

[19] Yang C. (2006). Evaluating latent class analyses in qualitative phenotype identification. Comput Stat Data Anal.

[20] Antonovsky A. (1993). The structure and properties of the sense of coherence scale. Soc Sci Med.

[21] Bao L., Liu J. (2005). The reliability and validity of Chinese version of SOC-13. Chin J Clin Psychol.

[22] Scheier M.F., Carver C.S., Bridges M.W. (1994). Distinguishing optimism from neuroticism (and trait anxiety, self-mastery, and self-esteem): a reevaluation of the life orientation test. J Pers Soc Psychol.

[23] Lai J.C.L., Cheung H., Lee W. (1998). The utility of the revised life orientation test to measure optimism among Hong Kong Chinese. Int J Psychol.

[24] Rosenberg M. (1965).

[25] Shen Z., Cai T. (2008). Disposal to the 8th item of Rosenberg Self-Esteem Scale(Chinese Version). Chin Ment Health J.

[26] Wang S., Zhang Z., Liu X. (2016). Test with the inner peace state scale in college students. Chin Ment Health J.

[27] Mccullough M.E., Emmons R.A., Tsang J.A. (2002). The grateful disposition: a conceptual and empirical topography. J Pers Soc Psychol.

[28] Wei C., Wu H., Kong X. (2011). Revision of gratitude Questionnaire-6 in Chinese adolescent and its validity and reliability. Chin J School Health.

[29] Xiao S. (1994). The theoretical basis and research application of social support rating scale. J Clin Psychiatr.

[30] Liu Q., Ge R., Zhu Y. (2024). The potential characteristics of the sense of coherence in cancer radiotherapy patients and its correlation with coping strategies. Support Care Cancer.

[31] Wang H., Wu Y., Huang X., Yan H. (2025). Relationship between sense of coherence and subjective well-being among family caregivers of breast cancer patients: a latent profile analysis. Front Psychiatr.

[32] Zhang X., Huang T., Sun D. (2025). Illness perception and benefit finding of thyroid cancer survivors: a chain mediating model of sense of coherence and self-disclosure. Cancer Nurs.

[33] Kissane D.W., Bobevski I., Appleton J. (2023). Meaning and Purpose (MaP) therapy in advanced cancer patients: a randomised controlled trial. Support Care Cancer.

[34] van Dongen S.I., de Nooijer K., Cramm J.M. (2020). Self-management of patients with advanced cancer: a systematic review of experiences and attitudes. Palliat Med.

[35] Ma H., Hu K., Wu W. (2024). Illness perception profile among cancer patients and its influencing factors: a cross-sectional study. Eur J Oncol Nurs.

[36] Leonhart R., Tang L., Pang Y. (2017). Physical and psychological correlates of high somatic symptom severity in Chinese breast cancer patients. Psychooncology.

[37] Verduzco-Aguirre H.C., Babu D., Mohile S.G. (2021). Associations of uncertainty with psychological health and quality of life in older adults with advanced cancer. J Pain Symptom Manag.

[38] Neel C., Lo C., Rydall A. (2015). Determinants of death anxiety in patients with advanced cancer. Bmj Support Palliat.

[39] Hinz A., Schulte T., Ernst J. (2023). Sense of coherence, resilience, and habitual optimism in cancer patients. Int J Clin Hlth Psyc.

[40] Ciria-Suarez L., Calderon C., Fernández Montes A. (2021). Optimism and social support as contributing factors to spirituality in cancer patients. Support Care Cancer.

[41] Cheng Q., Chen Y., Duan Y. (2024). Exploring the spiritual needs of patients with advanced cancer in China: a qualitative study. Sci Rep.

[42] Sun D., Zhang X., Cui M. (2023). Association between self-esteem and fear of cancer recurrence in cancer survivors: a cross-sectional study. Eur J Oncol Nurs.

[43] Cecon N., Pfaff H., Lee S. (2021). A salutogenic model predicting the need for psycho-oncological care and its utilisation-the role of generalized resistance resources and sense of coherence. Eur J Cancer Care.

[44] Tian X., Zhou X., Sun M. (2024). The effectiveness of positive psychological interventions for patients with cancer: a systematic review and meta-analysis. J Clin Nurs.

[45] Harrop E., Noble S., Edwards M. (2017). Managing, making sense of and finding meaning in advanced illness: a qualitative exploration of the coping and wellbeing experiences of patients with lung cancer. Sociol Health Illness.

[46] Folkman S., Lazarus R.S., Gruen R.J., DeLongis A. (1986). Appraisal, coping, health status, and psychological symptoms. J Pers Soc Psychol.

